# PEDOT:PSS Morphostructure and Ion-To-Electron Transduction and Amplification Mechanisms in Organic Electrochemical Transistors

**DOI:** 10.3390/ma12010009

**Published:** 2018-12-20

**Authors:** Pasquale D’Angelo, Giuseppe Tarabella, Agostino Romeo, Simone Luigi Marasso, Alessio Verna, Matteo Cocuzza, Carlotta Peruzzi, Davide Vurro, Salvatore Iannotta

**Affiliations:** 1 Istituto dei Materiali per l’Elettronica ed il Magnetismo, IMEM-CNR, Parco Area delle Scienze 37/A, 43124 Parma, Italy; romago23@gmail.com (A.R.); matteo.cocuzza@infm.polito.it (M.C.); carlotta.peruzzi@hotmail.it (C.P.); davide.vurro@imem.cnr.it (D.V.); salvatore.iannotta@imem.cnr.it (S.I.); 2 Camlin Italy Srl, Strada Budellungo 2, 43123 Parma, Italy; g.tarabella@camlintechnologies.com; 3 Chilab, Materials and Microsystems Laboratory, Department of Applied Science and Technology (DISAT), Politecnico di Torino, Via Lungo Piazza d’Armi 6, 10034 Chivasso (Torino), Italy; Alessio.Verna@polito.it

**Keywords:** OECT, PEDOT:PSS, biosensor, bioelectronics

## Abstract

Organic electrochemical transistors (OECTs) represent a powerful and versatile type of organic-based device, widely used in biosensing and bioelectronics due to potential advantages in terms of cost, sensitivity, and system integration. The benchmark organic semiconductor they are based on is poly(3,4-ethylenedioxythiophene):polystyrene sulfonate (PEDOT:PSS), the electrical properties of which are reported to be strongly dependent on film morphology and structure. In particular, the literature demonstrates that film processing induces morphostructural changes in terms of conformational rearrangements in the PEDOT:PSS in-plane phase segregation and out-of-plane vertical separation between adjacent PEDOT-rich domains. Here, taking into account these indications, we show the thickness-dependent operation of OECTs, contextualizing it in terms of the role played by PEDOT:PSS film thickness in promoting film microstructure tuning upon controlled-atmosphere long-lasting thermal annealing (LTA). To do this, we compared the LTA-OECT response to that of OECTs with comparable channel thicknesses that were exposed to a rapid thermal annealing (RTA). We show that the LTA process on thicker films provided OECTs with an enhanced amplification capability. Conversely, on lower thicknesses, the LTA process induced a higher charge carrier modulation when the device was operated in sensing mode. The provided experimental characterization also shows how to optimize the OECT response by combining the control of the microstructure via solution processing and the effect of postdeposition processing.

## 1. Introduction

Based on their proven biocompatibility and their ideal amplifying interface between ionic and electronic signals, organic electrochemical transistors (OECTs) have shown a marked suitability for studying phenomena depending on fluxes of specific ions. For instance, OECTs have been successfully employed for studying the function of cell membranes [1] and biosystems in general [2,3,4], but also for studying molecular interactions (e.g., molecular recognition [5] or enzyme-catalyzed chemical reactions [6]). This is because they combine a recognition system in which chemical reactions involved in biological processes take place with the ion-to-electron transduction capability of biocompatible, conformable organic conductors (OCs). The conformable character of the flexible polymeric materials makes the related devices appropriate for interfacing with living beings [7] and the manufacturing of implantable devices [8,9]. In addition, the transistor-like architecture of OECTs promotes device integration (of interest for consumer electronics) and allows the implementation of multifunctional devices (of interest for neuromorphic applications [10,11]) and disposable lab-on-a-chip (LOC) or standard biological platforms for the study of biological processes and biomarkers [12,13,14,15].

OECT operation relies on an efficient reduction of the oxidized p-type doped Poly(3,4-ethylenedioxythiophene):polystyrene sulfonate (PEDOT:PSS) film, acting as the device channel, and is promoted by charged species of different nature and size [16,17] that are forced from an electrolytic reservoir toward the channel by an electric field applied between the gate electrode and the device channel. The interaction between the charged species and the film bulk leads to the modulation of the current flowing through the device channel. This process is favored by the negligible barrier effect for charged species crossing the electrolyte–OC interface, as reported by Rivnay et al. [9]. It has been also demonstrated that for OECTs the transconductance (*g*_m_) is higher than for most solid-state devices [18], showing a correct operation even at zero gate bias [19].

PEDOT:PSS is the benchmark material within the class of conducting electroactive organics for OECT applications [20]. This compound is constituted by a stable conjugated polymer (PEDOT), the water dispersiblity of which is promoted by the addition of a water-soluble polyelectrolyte (PSS), acting also as the charge-balancing counter ion during PEDOT:PSS polymerization [21]. In addition, PSS plays the role of a primary dopant agent for PEDOT, and hence PEDOT:PSS films show an efficient p-type conduction [22]. PEDOT:PSS in aqueous solution promotes the formation of micelle, as the hydrophobic polycationic PEDOT chains tend to form dispersed grains surrounded by the polyanionic PSS chains, attached on the PEDOT clusters via their sulfonate groups [23,24]. The film, achieved by means of solution processing techniques, replicates the typical features of the aqueous suspension. Hence, depending on the film post processing, the thin film morphology reveals a segregation of the PEDOT phase to different extents. In detail, the segregation process acts on the redistribution of the granular structures made of PEDOT clusters in the polymeric matrix made of an excess of weakly ion-conducting PSS [21,25] in which such structures are embedded.

During the last two decades, the basic interest of researchers has been focused upon the possible effect of some peculiar morphostructural changes of PEDOT:PSS on its physical properties. A set of related studies have demonstrated morphological rearrangements induced by conformational changes in the PEDOT chain, with a net impact on phase segregation [26,27]. The solvent annealing (or secondary doping procedure) operated by using co-solvents such as ethylene glycol (EG) [28] and dimethyl sulfoxide (DMSO) [29] is known to reduce the excess of PSS via an enhanced phase segregation process and, as a consequence, to increase the conductivity by a few orders of magnitude [30]. The conformational change of the PEDOT chains from coiled to linear (or expanded-coil) [23] results in a lengthening of PEDOT-rich clusters along the in-plane direction and a constriction of PSS insulating barriers. This process strongly depends on the film processing as, for instance, the spinning speed during the deposition by the spin-coating technique affects the thickness and surface quality [31], whereas even the composition of the thin film is sensitive to the deposition conditions. The promotion of an efficient phase segregation has been associated with the annealing procedure, which is known to act on the excess of PEDOT:PSS [32,33]. Structural features accompany conformational changes as well, since the quinoid form of PEDOT chains is preferred in the case of the expanded-coil structure, whereas the coil conformation with thicker PEDOT clusters favors the benzoid form [26], which is predominant in the case of undoped or very thin films [34].

Strong implications on film conductivity have been highlighted by numerous works correlating PEDOT:PSS processing to electrical, structural, and morphological studies. Temperature-dependent analysis of electrical conduction has enlightened a strong anisotropy in conductivity, showing how the in-plane transport can be associated with a non-nearest-neighbor hopping, such as 3D variable range hopping (VRH), for films made of the pristine material [25]. A more efficient quasi-1D-VRH favored by an enhanced extent of the phase segregation process is promoted by solvent annealing [35,36]. On the contrary, due to a larger vertical separation between adjacent PEDOT-rich domains with respect to their in-plane connectivity, the Arrhenius-like thermally activated process indicates that a nearest-neighbor hopping (nn-H) rules the out-of-plane conductivity in solvent annealed films [35]. These experimental findings have been corroborated by scanning probe microscopy (SPM) studies, where the features of phase segregation have been associated with a solvent annealing-induced inhomogeneity of the out-of-plane distribution of PEDOT clusters, specifically in terms of their size and thickness-dependent content [32,37].

Beyond these basic features, there is a marked sensitivity of PEDOT:PSS to multifarious factors influencing the thin film physical properties. It has been reported, for instance, that the solvent annealing induces an increase of red-ox active charge carriers, thus widening the conductive potential window [38], and that both the quinoid and benzoid structures can exist with a different relative weight in solvent-annealed [39] and undoped films [40].

An understanding of the strong dependence of PEDOT:PSS thin film physical properties on material processing importantly guides the way for an efficient control of PEDOT:PSS structural features and thin film morphology, but also demonstrates that the control of the related electronic devices operation requires careful attention to these issues.

In light of the depicted scenario, in this work we studied the thickness-dependent response of OECTs post-processed by long-lasting thermal annealing (LTA), focusing the device response analysis on the competition between PEDOT:PSS morphological features and OECT operation mechanisms. This was done by comparing the LTA-OECT response to that of OECTs post-processed by rapid thermal annealing (RTA) (i.e., a fast film drying). In detail, we took advantage of the cues offered by the literature in order to induce a significant inhomogeneity of both PEDOT cluster distribution and size within the bulk of PEDOT:PSS thin films along the out-of-plane axis, also within the limit of very low film thicknesses. As reported in the literature, such inhomogeneity can be induced by exposing the as-deposited PEDOT:PSS films to the LTA process [31] carried out under controlled environmental conditions [41]. Our aim was to evaluate the role of the thickness on the LTA effect by analyzing the performance of our set of devices. The device performance was assessed under different biasing conditions and compared to that of devices exposed to the RTA process, which was expected to poorly affect the vertical separation of PEDOT grains [31]. We clarify relevant aspects of how to control PEDOT:PSS features that are critical to OECT performance, and hence we clarify how to develop an optimization of the processes.

## 2. Materials and Methods

### 2.1. Chemicals

Water dispersion of PEDOT:PSS (Clevios PH1000) was purchased from Heraeus (Holding GmbH Hanau, Germany). Ethylene glycol (EG), isopropyl alcohol (IPA), dodecyl benzene sulfonic acid (DBSA), 3-glycidoxypropyltrimethoxysilane (GOPS), NaCl in powder, and Sylgard 184 poly(dimethylsiloxane) (PDMS) were purchased from Sigma Aldrich (St. Louis, MO, USA). Silicon (Si) wafers with a 1µm-thick thermally grown silicon dioxide (SiO_2_) layer were purchased from Agar Scientific Ltd. (Stansted, UK). Milli-Q water (resistivity > 18.2 MΩ) was used to prepare the NaCl electrolyte solutions. Silver wires, used as gate electrodes, were purchased from Franco Corradi Srl (Rho, Italy).

### 2.2. Device Fabrication

**Figure 1:**
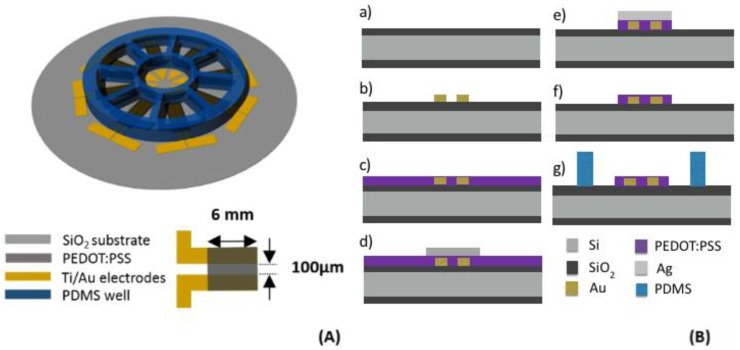
(**A**) Organic electrochemical transistor (OECT) layout. (**B**) Device fabrication process: (a) SiO_2_ substrate, (b) patterned electrical contacts, (c) poly(3,4-ethylenedioxythiophene):polystyrene sulfonate (PEDOT:PSS) film, (d) Ag layer, (e) excess of PEDOT:PSS removed by O_2_ plasma, (f) Ag removal by E6 wet etching, and (g) bonding of poly(dimethylsiloxane) (PDMS) well.

The device was developed starting from a 2-inch circular-shaped single-side-polished Si wafer having a very flat SiO_2_ layer (surface roughness below 1 nm) on both sides (Figure 1A,B). After a preliminary cleaning with acetone and isopropanol (IPA), the source, drain, and gate electrodes made of a Ti (20 nm)/Au (100 nm) bilayer were deposited together with the related contact pads by using a Kurt J. Lesker (Jefferson Hills, PA, USA) PVD 75 DC Magnetron Sputtering. The Ti/Au bilayer was patterned by a lift-off technique based on a commercial image reversal AZ 5214E photoresist and a dimethyl sulfoxide bath at 60 °C (Figure 1B(b)). The process allowed for obtaining eight patterns with a 100-µm-long and 6-mm-wide channel (Figure 1A), resulting in a channel area of 0.6 mm^2^ and an aspect ratio = 60. After that, PEDOT:PSS films were deposited on the substrate by spin-coating the aqueous suspension pretreated with EG (for secondary doping), GOPS (for improving film stability and adhesion), and DBSA (for film forming) [42]. In this way, a set of 6 samples with thicknesses of 15, 20, 25, 35, 70, and 130 nm was obtained. The spun films were heat-treated at 150 °C in vacuum for 90 min in order to promote the phase segregation process via an LTA process under controlled environmental conditions [41] (Figure 1B(c)). Silver (Ag) was chosen as an etch mask in order to protect PEDOT:PSS films [43], so a 150-nm-thick Ag layer was deposited using an ULVAC EBX-14D electron beam evaporator (ULVAC, Chigasaki, Japan). The Ag layer was subsequently patterned by a wet etching involving the commercial solution E6 Metal Etching (16 vol H_3_PO_4_ (85%)/1 vol HNO_3_ (65%)/1 vol CH_3_COOH/2 vol H_2_O) at room temperature (Figure 1B(d)). A commercial positive-tone photoresist HPR 504 was used for the photolithography step, whereas the etching process was completed by photoresist stripping with acetone. After the definition of the channel region, the PEDOT:PSS excess was removed using an O2 plasma (100 mTorr, 50 sccm, 100 W, 13.56 Hz) for 30 s (Figure 1B(e)), and the residual Ag layer, involved in the protection of PEDOT:PSS, was finally removed by the aforementioned E6 wet etching (Figure 1B(f)). Finally, a PDMS reservoir fabricated by soft lithography and 100 µL in volume was placed in correspondence with each channel region through the stick-and-stamp technique (Figure 1B(g)) with the aim of confining the liquid electrolyte at the interface with the device channel. 

A second batch of samples were fabricated, modifying the PEDOT:PSS post-processing step for three benchmark thicknesses of 50, 100, and 200 nm. In this case, the devices were post-baked following an RTA process (i.e., at 180 °C for 10 min), aimed only at inducing a fast dry of the polymer.

### 2.3. OECT Measurements

Conductivity values were extracted from current-voltage measurements carried out using a homemade four-point probe resistivity measurement system connected to a 2-channel source-meter precision unit (B2902A, Agilent, Santa Clara, CA, USA).

OECT measurements were recorded using the same electrometer controlled by a customized LabView V.5.1 software (National Instruments, Austin, TX, USA) able to record simultaneously both the channel (*I*_ds_) and gate (*I*_gs_) currents. Prior to any measurement, the OECT channel was exposed to Milli-Q water for 10 min in order to properly hydrate the conducting polymer and stabilize its conductive properties [42]. The excess of PSS was expected to be accordingly removed [44], and swelling issues were basically minimized during the measurement [45]. All electrical measurements were carried out using a NaCl solution (0.15 M in Milli-Q water) as the electrolyte and an Ag wire as the gate electrode. 

**Figure 2:**
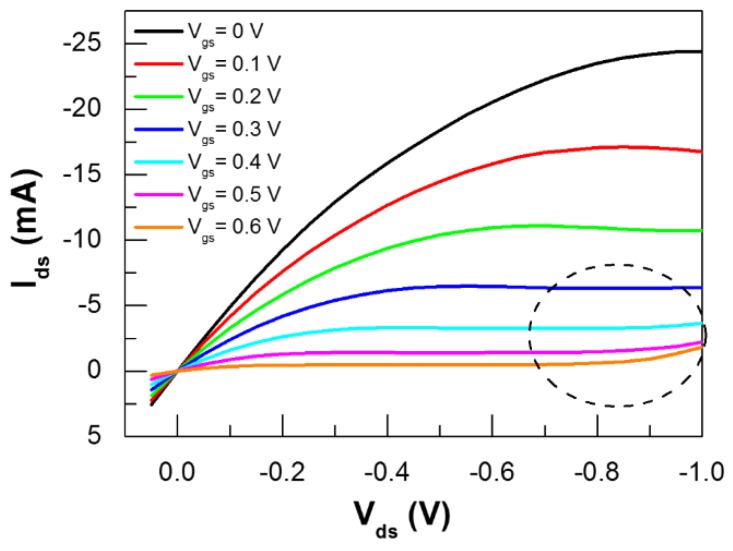
Output characteristics of an OECT with a 70-nm-thick PEDOT:PSS channel recorded by sweeping *V*_ds_ between 0.05 V and −1 V at a scan rate of 50 mV/s and a constant gate bias (*V*_gs_) fixed between 0 and 0.6 V, with *V*_gs_ steps of 0.1 V. The dotted circle corresponds to the onset of the electrolysis of the aqueous sodium chloride electrolyte.

Output characteristics were recorded by sweeping the source-drain voltage (*V*_ds_) between −0.05 and 1 V at a scan rate of 50 mV/s and at a constant gate bias (*V*_g*s*_), and then repeating each channel voltage sweep at different fixed *V*_gs_ values comprised of between 0 and 0.6 V (*V*_gs_ steps of 0.1 V). Transfer characteristics were recorded by sweeping *V*_gs_ between −0.5 and 0.6 V and at different fixed *V*_ds_ values, with a scan rate of 10 mV/s (Figure 2).

The evaluation of the current modulation parameter Δ*I*/*I*_0_ (expressing the OECT transducing capability) was performed by measuring the I_ds_ versus time curve using a constant source-drain voltage and pulsing the gate voltage between 0 V and a positive value [42]. In this study, we fixed *V*_ds_ at −0.6 V and pulsed *V*_gs_ between 0 and three different values of 0.05 V, 0.2 V, and 0.4 V, with steps lasting 20 s.

Three different devices were analyzed for each thickness, and modulation results were weight-averaged over all the calculated values. The device was biased by choosing different sets of the two biasing voltages in a way that their additional contribution remained below the electrolysis voltage of aqueous sodium chloride (|*V*_gs_|+|*V*_ds_| < 1.23 V [46], see Figure 2). In this way, we prevented the possible interaction of the PEDOT:PSS interface with gaseous hydrogen [47].

### 2.4. Atomic Force Microscopy (AFM) Measurements

For these, 3 × 3 µm^2^ AFM topography was acquired in tapping mode by using a Dimension 3100 Scanning Probe Microscope equipped with a Nanoscope IVa controller (Veeco Instruments, Plainview, NY, USA). Statistical analysis of AFM micrographs was performed by using the NanoScope 8.15 Software (Veeco instruments).

## 3. Results

**Figure 3:**
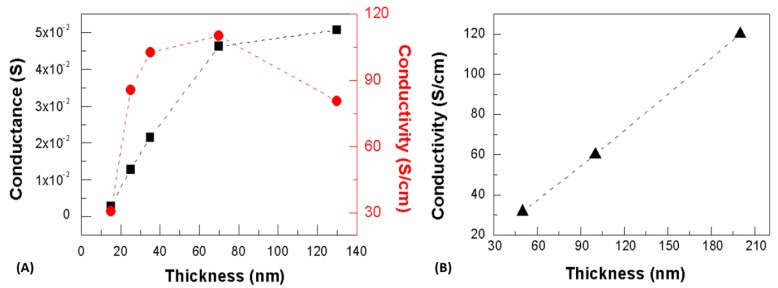
(**A**) Conductance (black squared symbols) and conductivity (red circles) as a function of the device channel thickness for long-lasting thermal annealing (LTA) processing; (**B**) Conductivity versus channel thickness for films processed under rapid thermal annealing (RTA) conditions.

The controlled-atmosphere post-processing conditions adopted for LTA films were expected to induce an efficient reorientation of the PEDOT:PSS clusters [31,41]. The impact of the LTA post-processing on the intrinsic charge carrier transport was evidenced by the electrical characterization reported in Figure 3A. The conductivity versus thickness curve showed an increase and saturating behavior for thicknesses between 15 and 70 nm followed by a significant conductivity reduction of about 30% at 130 nm. The charge transport for solvent annealed films was promoted by an in-plane quasi one-dimensional Variable Hopping Range (VHR) conduction mechanism, and a thickness-dependent conductivity enhancement was expected because of an efficient out-of-plane interconnection of PEDOT grains promoting a long-range connectivity for conductive clusters [48,49]. For thicker films, this long-range connectivity can be influenced by a prominent vertical separation of grains causing a strong out-of-plane inhomogeneity and therefore leading to a less efficient in-plane transport, as occurs for pristine PEDOT:PSS films characterized by a three dimensional VHR-dominated transport [50].

RTA processing promotes a fast dry and insufficient time for an efficient rearrangement of PEDOT and PSS phases, thus resulting in a weak segregation [31,35]. The extent of percolative pathways is limited accordingly [48], as a fast dry is not able to promote either a substantial rearrangement of PEDOT and PSS phases, nor the coalescence of smaller grains into larger ones [31]. The conductivity versus thickness curve reported in Figure 3B shows the linear behavior already shown for films processed under similar conditions [51]. Preliminary tests on air-dried films showed conductivity values comparable to those of RTA-treated films. In this respect, conductivity values were lower than those assessed for films processed by a controlled annealing due to a less efficient packing of PEDOT grains that turned out in a pronounced diphasic character exhibited by PEDOT:PSS films post-processed by a rapid annealing procedure.

The effect of the early-stage segregation process on the sensing response and amplification of PEDOT:PSS-based OECTs was evaluated upon different combinations of the device biasing voltages *V*_ds_ and *V*_gs_.

**Figure 4:**
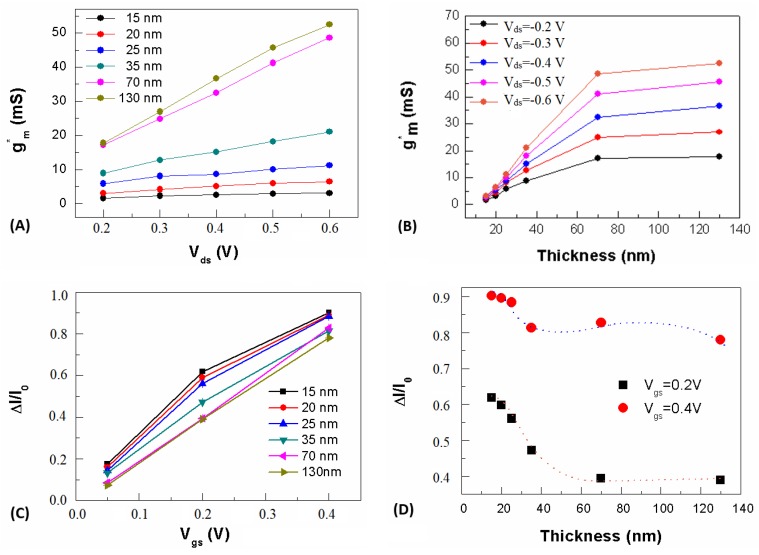
(**A**) OECT transconductance *g*_m_ as a function of channel biasing voltage *V*_ds_ at different PEDOT:PSS thicknesses; (**B**) *g*_m_ as a function of channel thickness for *V*_ds_ values comprised between 0.2 and 0.6 V, with *V*_ds_ steps of 0.1 V; (**C**) current modulation parameter Δ*I*/*I*_0_ as a function of *V*_gs_ at different PEDOT:PSS thicknesses and (**D**) as a function of thickness at *V*_gs_ = 0.2 V (black squares) and *V*_gs_ = 0.4 V (red circles) (orange and blue dotted lines are just for guiding the eye).

Here, *g*_m_ versus *V*_gs_ curves were extracted from typical transfer curves *I*_ds_ versus *V*_gs_, as described in Supplementary Materials Section (Figure S1). The impact of the device features on the transducing and amplifying operation was enlightened by evaluating the dependence of both transconductance peaks *g*_m_* and the Δ*I*/*I*_0_ parameter on the OECT biasing conditions (Figure 4A) and active channel thickness (Figure 4B), respectively. As a result, *g*_m_* linearly increased with *V*_ds_, and the dependence on *V*_ds_ was marked for higher thicknesses up to the “threshold” thickness of 70 nm, where a crossover toward a saturating behavior took place (Figure 4A). The saturation of *g*_m_* for channels thicker than 70 nm was remarked by slight variations of *g*_m_* for large variations of film thickness (Figure 4B).

Recently, the “volumetric capacitance (*C*_vol_)” of the PEDOT:PSS thin film has been demonstrated to be a key property of an efficient OECT amplification operation [18]. In particular, an enhancement of *g*_m_ was promoted by a scaling of *C*_vol_ with the film volume [9]. The origin of *C*_vol_ has been attributed by Proctor et al. to the presence of sites consisting of sulfonate anion and hole pairs acting as capacitive elements [52]. Then, cations injected from the electrolyte into the film bulk tend to replace holes (intrinsic charge carriers for PEDOT:PSS), giving rise to an enhanced capacitance per unit volume. The proposed model describes these pairs as distributed within the film bulk and spaced by a distance α expressing the average distance between adjacent pairs. Within this scheme, the redistribution of the two phases in the compound (i.e., PEDOT clusters and PSS excess) and their specifications in terms of PEDOT cluster size and PSS barrier thickness are expected to influence the mechanism of interaction between injected cations and sulfonate anion and hole pairs. The saturating behavior that we observed above 70 nm indicated that the volumetric capacitance could be strongly sensitive to the morphological changes induced by the thickness-dependent LTA process, rather than to structural changes of PEDOT chains. This was because the quinoid phase was expected to dominate for EG-doped films [26]. Additionally, similar morphological features observed for thicker films suggested that the vertical separation was sensitive to the film thickness and more effective on the PSS excess rather than on the promotion of thinner PSS shells accompanying the elongation of PEDOT clusters.

A saturating trend was found for LTA films, whereas upon RTA an increase of *g*_m_* over the whole spanned thickness range emerged (see Figure S2) [9,51]. Nevertheless, again *g*_m_* shared values higher in the case of LTA post-processing.

These results indicated that two competitive factors concurred at determining the OECT response. On the one hand, the better packing of PEDOT grains led to an enhancement of *g*_m_* correlated to the decrease of the average distance between adjacent PEDOT hole and PSS sulfate anion pairs. On the other hand, the enhanced vertical phase segregation occurring at higher thicknesses led to a saturation of *g*_m_* that could be explained in terms of the statistical increase of the average distance between the same pairs induced by the out-of-plane inhomogeneity.

The *g*_m_* versus *V*_gs_ trend at the lowest thickness, reported in Figure S1D, showed that the transconductance peak above 100-µm-long channels was achieved for gate biasing next to zero volts, similarly to what has been reported elsewhere for similar devices in terms of channel length [19]. This indicated that the spontaneous diffusion at lower thicknesses, favored by the negligible potential barrier for ionic adsorption by PEDOT:PSS, induced an effective uptake of ions over the whole bulk of PEDOT:PSS films. On the contrary, the highest transconductance value for higher thicknesses required a higher driving force for cationic injection into the PEDOT:PSS thick films. Hence, by increasing the film thickness, the peak position turned out to be located at a higher *V*_gs_, even if *g*_m_* was insensitive to thicknesses larger than 70 nm. Interestingly, a double peak emerged for thinner films, so an efficient amplification of ionic signals could be obtained even for the negative gate biasing region, where the device operation was not related to cations uptake but rather to a recovery of the intrinsic hole conduction of the PEDOT:PSS film.

**Figure 5:**
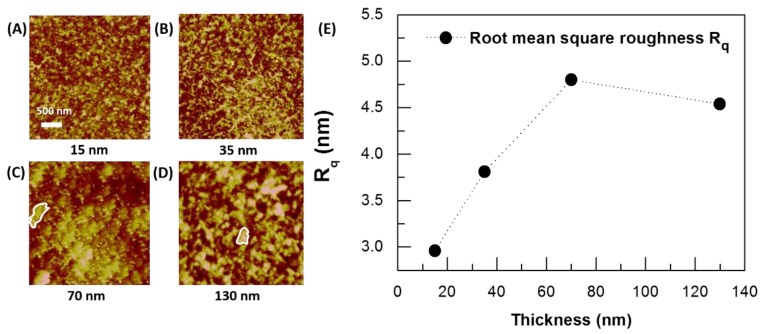
AFM micrographs for (**A**) 15-, (**B**) 35-, (**C**) 70-, and (**D**) 130-nm-thick PEDOT:PSS films (scale bar 500 nm; white circles indicate typical PEDOT domains); (**E**) saturated root mean square roughness (*R*_q_) as a function of the device channel thickness.

*C*_vol_ was demonstrated to be effective also in terms of the channel current modulation (transducing capability) for devices processed in a similar way and operated by using different liquid electrolytes [51]. Therefore, the modulation parameter Δ*I*/*I*_0_ versus *V*_gs_, reported in Figure 4C, was thickness-dependent in the range of thicknesses where a dynamic morphological change was found to have taken place, as shown by scanning probe microscopy studies [29,32]. Conversely, Δ*I*/*I*_0_ showed a saturating trend in the region of higher thicknesses (Figure 5D). A saturation of Δ*I*/*I*_0_ was ideally expected in the lower thickness range, too, as the thinnest device channel one could imagine comprised at least a single monolayer of interconnected PEDOT:PSS clusters. In the case of RTA films, Δ*I*/*I*_0_ decreased monotonically over the investigated thickness range, whereas the modulation was less effective at higher thicknesses (Figure S2B).

The role of PEDOT:PSS post-processing on the OECT switching speed was also studied by evaluating the device response time (τ_res_) following the procedure described in the Supplementary Materials file (see Figure S3A). We found a slight dependence of τ_res_ on *V*_gs_ (Figure S3B) that was more pronounced at the highest thickness and (see Figure S3C,D) a monotonic increase with the film thickness [18]. In contrast to the very high *g*_m_* assessed for our devices (falling in the range 1.5–52 ms), the assessed response times (ranging between tens of milliseconds and about 270 ms) underperform those found in the case of miniaturized devices with aspect ratios ranging from 1 to 2, showing a τ_res_ falling in the range 10–100 µs [18,53]. A device with a downscaled channel area determines a better coupling between the gate electrode and the device channel, thanks to a more favorable gate-to-channel area ratio [54] that induces an efficient dynamic change of channel resistivity [54,55]. This explains why devices with longer and wider channels have shown even higher response times compared to those assessed in this study [51]. In addition, device response times observed for RTA post-processed films (not reported) showed an order of magnitude similar to those found for LTA films (τ_res_ ranged between 70 and 390 ms). Therefore, the device switching speed seemed to not be affected by changes induced on film morphology by post-processing, but it could rather have depended on the device channel specific characteristics.

The above analysis suggests that the OECT response may have depended on PEDOT:PSS thickness-sensitive morphological bulky changes due to PEDOT grains coalescence and related phase segregation, a process driven by the controlled post-processing procedure.

In the following, AFM analysis was used as a complementary support to objective verification of what has been reported in the literature. Specifically, AFM characterization performed by Wang et al. has indicated that an enhanced phase segregation in PEDOT:PSS corresponds to a higher surface roughness, which has been reported to be also thickness dependent [56]. In our case, AFM micrographs, reported in Figure 5 for 15-, 35-, 70-, and 130-nm-thick LTA films, showed some round-shaped bright domains randomly distributed on the film surface. Their surface distribution and size both increased with the film thickness, up to 70 nm. Then, a less marked difference was observed going from the 70-nm to the 130-nm-thick film (Figure 5C,D). These large granular domains have been associated by several reports with high-conducting PEDOT clusters with an enhanced size promoted by solvent annealing [29,32,57]. A relevant enhancement of vertical phase segregation is expected as a function of film thickness, too, together with a lateral widening of grain size [34]. On this basis, the grain-like structure that emerged for thicker films could be seen as a fingerprint of a thickness-dependent phase segregation process.

Keeping in mind the indications outlined in Reference [56], we made a statistical analysis of the surface topographies shown in Figure 5A–D. This was done by evaluating the root mean square roughness *R*_q_, a parameter expressing the measure of the topographical statistical fluctuations around an average surface height. Due to its length-scale invariant character, *R*_q_ can mirror the bulky redistribution of the two phases of diphasic polymeric blends and compounds upon thermally induced phase segregation and the related small grains coalescence [58,59]. The evaluated *R*_q_, reported as a function of thickness in Figure 5E, grew for increasing thicknesses of up to 70 nm, showing an almost saturated behavior above 70 nm. The upscaling of *R*_q_ between 15 and 70 nm could be correlated with the lateral widening of grains observed upon increasing the PEDOT:PSS film thickness [57]. The grain size widening was then favored by the phase segregation enhancement, which resulted in an improved coalescence of conductive PEDOT-based clusters that gave rise to hundreds nanometers-sized domains (Figure 5C,D). Hence, the saturation trend of *R*_q_ above 70 nm may indicate an equilibrium condition for both the in-plane PEDOT grain coalescence and the spatial redistribution of clusters along the vertical direction. It emerged that an effective vertical separation induced by post-processing took place upon LTA post-processing, even at relatively low thicknesses.

## 4. Conclusions

In this work, we studied in detail the important role played by PEDOT:PSS thickness, a key parameter for the optimization of OECT operation. The reported results give a better insight into the effects of fabrication processes on PEDOT:PSS morphological features. Our results showed that, for devices with high aspect ratios (60), larger thicknesses corresponded with higher gains for OECTs working as switches in a transistor-like mode (52 ms at *V*_gs_ = 0.6 V for 130-nm-thick LTA film), whereas smaller thicknesses provided a higher charge carrier modulation (up to 0.89 for 20-nm-thick LTA film), as required for proper operation in sensing mode. Nevertheless, all our experimental findings, if correlated to literature studies on the tuning of PEDOT:PSS morphostructural properties upon film processing, confirmed the active role played by film morphology in terms of OECT operation. In particular, we verified how LTA post-processing realized an optimal redistribution of the multiple interfaces within the as-deposited PEDOT:PSS-based OECT channels. Accordingly, a better intrinsic PEDOT:PSS conduction, but also a high gain and a marked transducing capability of related OECTs, were obtained upon LTA processing, until both the dynamics of small PEDOT:PSS grains coalescing into larger clusters saturated and the out-of-plane segregation process was completed. Finally, morphological changes did not affect the assessed device response times (ranging between tens of milliseconds and a few hundreds of milliseconds, in the worst case), which rather depended on the device channel specifications.

## Supplementary Materials

The following are available online at http://www.mdpi.com/1996-1944/12/1/9/s1, Figure S1: Typical transfer characteristics *I*_gs_ versus *V*_gs_ for the OECT with (A) 15 nm, (B) 70 nm, and (C) 130 nm thick channels. Transconductance curves *g*_m_ versus *V*_gs_ for (D) 15 nm, (E) 70 nm, and (F) 130 nm thick channels, Figure S2: (A) Transconductance peak *g*_m_* versus thickness at *V*_ds_ = 0.4 V for films processed by an RTA post-processing procedure; (B) effect of the post-processing conditions on the current modulation parameter Δ*I*/*I*_0_, reported as a function of PEDOT:PSS thickness, for films processed by RTA (black squares) and LTA (red circles) post-processing procedures (orange and blue dotted lines are guides for the eye), Figure S3: (A) Typical modulation step (black curve) recorded at *V*_ds_ = −0.1 V, *V*_gs_ = −0.4 V, and a double exponential decay fit curve (red curve); (B) device response time τ_res_ versus *V*_gs_ for different device channel thicknesses at *V*_gs_ = 0.05, 0.1, and 0.4 V; τ_res_ versus channel thickness for (C) *V*_gs_ = 0.2 V and (D) *V*_gs_ = 0.4 V. References [9,60] are cited in the Supplementary Materials.
