# Supplementary material for: PEDOT:PSS Morphostructure and Ion-To-Electron Transduction and Amplification Mechanisms in Organic Electrochemical Transistors
Source: Materials (Basel). 2018 Dec 20;12(1):9. doi: 10.3390/ma12010009 (PMC6337112; doi:10.3390/ma12010009)
Supplement: Supplementary file 1 [file materials-12-00009-s001.pdf]

materials

MDPI

Supplementary Materials

OECT transconductance curves as a function of the gate bias (g_m versus V_{gs}) were calculated for all the thicknesses under study by differentiating the transfer curves ($g_m = \partial I_{ds}/\partial V_{gs}$) recorded for different channel voltages. A typical set of transfer curves, recorded by sweeping the gate bias between −0.2 V and 0.6 V (gate voltage scan rate of 10 mV/s) and fixing the drain voltage in the range between −0.2 and −0.6 V, with steps of −0.1V, is reported in Figure S1A–C for the thinnest (15 nm), intermediate (70 nm), and thickest (130 nm) films, respectively. The related g_m versus V_{gs} plot is reported in Figure S1D–F, respectively.

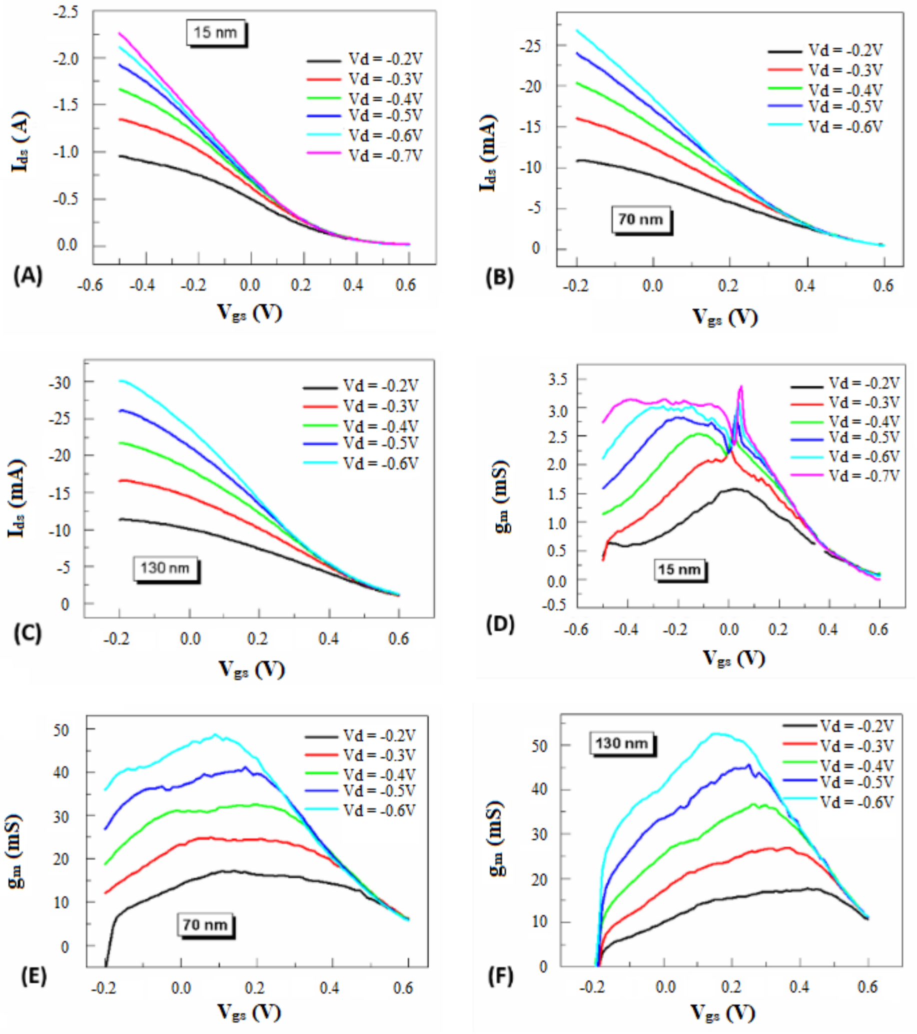

Figure S1. Typical transfer characteristics I_{gs} versus V_{gs} for the OECT with (**A**) 15 nm, (**B**) 70 nm, and (**C**) 130 nm thick channels. Transconductance curves g_m versus V_{gs} for (**D**) 15 nm, (**E**) 70 nm, and (**F**) 130 nm thick channels.

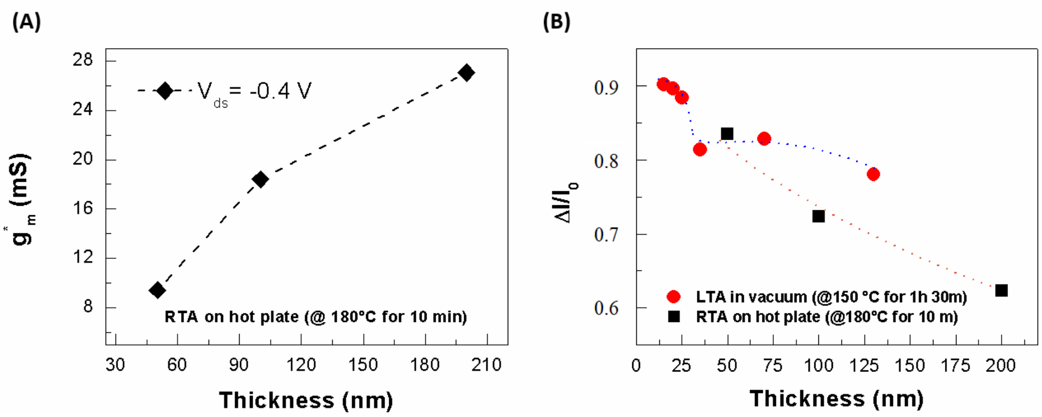

Figure S2. (**A**) Transconductance peak g_m* versus thickness at V_{ds} = 0.4 V for films processed by an RTA post-processing procedure; (**B**) effect of the post-processing conditions on the current modulation parameter $\Delta I/I_0$, reported as a function of PEDOT:PSS thickness, for films processed by RTA (black squares) and LTA (red circles) post-processing procedures (orange and blue dotted lines are guides for the eye).

OECT response times τ_{res} were calculated by recording three sets of time-dependent current modulation curves (I_{ds} versus time at a fixed channel biasing, V_{ds} = −0.1 V, for three step-like gate voltages of 0.1, 0.2, and 0.4 V, lasting 10 seconds) on LTA films [9].

I_{ds} versus time curve for 70-nm-thick film at V_{gs} = −0.4 V in Figure S3A is reported as an example. The best fitting curve for our experimental data was given by the superimposition of two exponential decays with different amplitudes of the pre-factor [60]. The higher time constant was of some seconds for all the samples, whereas the lower one, falling in the range between tens of milliseconds and about 270 ms for the analyzed devices, provided a faster exponential decay. In addition, the pre-factor of the faster exponential decay, lower by two or three orders of magnitude than that of the slower exponential decay, was comparable to the current versus time response related to the gate voltage step applied for measuring the time constant. This indicated that the long time constant determined the saturating de-doping effect quite after the gate potential turning on, whereas the lowest time constant accounted for the OECT switching time τ_{res}. The τ_{res} parameters were weight-averaged over all the calculated values for different thicknesses of LTA films (reported as a function of V_{gs} in Figure S3B). Figures S3C,D show the thickness-dependent enhancement of τ_{res}. Device response times were assessed for RTA post-processed films, too (not reported). We found a similar order of magnitude for response times of RTA films, even if in this case a more pronounced dependence of τ_{res} on gate voltage was found. In fact, for the 50-nm-thick device channel, τ_{res} ranged between 70 ms and 100 ms (for V_{gs} of 0.1 V and 0.4 V, respectively); for 100-nm-thick film, τ_{res} ranged between 100 ms (V_{gs} = 0.1 V) and 215 ms (V_{gs} = 0.4 V); whereas for 200-nm-thick film, τ_{res} ranged between 260 ms (V_{gs} = 0.1 V) and 390 ms (V_{gs} = 0.4 V).

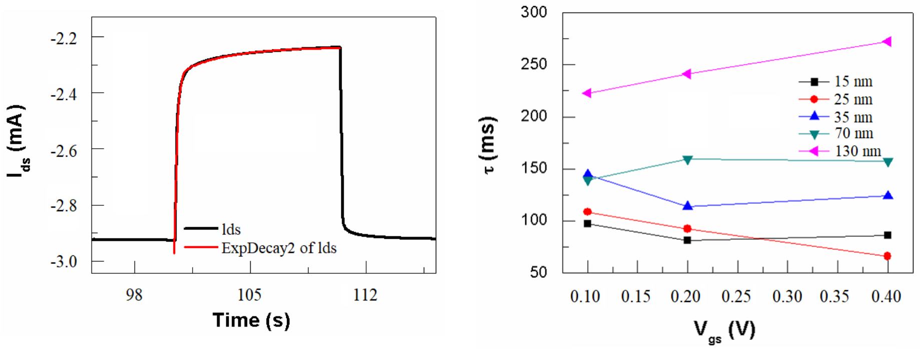

(**a**) (**b**)

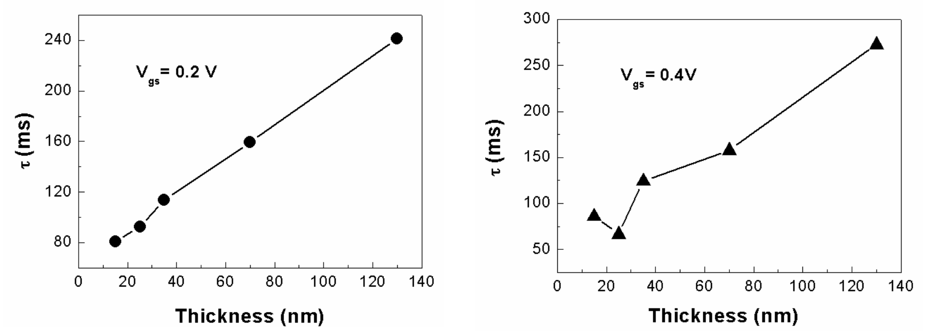

(**c**) (**d**)

Figure S3. (**A**) Typical modulation step (black curve) recorded at V_{ds} = −0.1 V, V_{gs} = −0.4 V, and a double exponential decay fit curve (red curve); (**B**) device response time τ_{res} versus V_{gs} for different device channel thicknesses at V_{gs} = 0.05, 0.1, and 0.4 V; τ_{res} versus channel thickness for (**C**) V_{gs} = 0.2 V and (**D**) V_{gs} = 0.4 V.
